# The catatonic dilemma expanded

**DOI:** 10.1186/1744-859X-5-14

**Published:** 2006-09-07

**Authors:** Heath R Penland, Natalie Weder, Rajesh R Tampi

**Affiliations:** 1 Department of Psychiatry, Yale University School of Medicine, New Haven, CT, USA

## Abstract

Catatonia is a common syndrome that was first described in the literature by Karl Kahlbaum in 1874. The literature is still developing and remains unclear on many issues, especially classification, diagnosis, and pathophysiology. Clinicians caring for psychiatric patients with catatonic syndromes continue to face many dilemmas in diagnosis and treatment. We discuss many of the common problems encountered in the care of a catatonic patient, and discuss each problem with a review of the literature. Focus is on practical aspects of classification, epidemiology, differential diagnosis, treatment, medical comorbidity, cognition, emotion, prognosis, and areas for future research in catatonic syndromes.

## Background

Catatonic syndromes embody the practice of psychiatry as well as any other psychiatric diagnosis. Catatonia presents commonly in psychiatric patients in both acute and long-term settings. Despite its common occurrence, catatonia remains a poorly understood, poorly studied, and poorly-recognized syndrome, presenting with a variety of psychiatric and medical illnesses, which can be treatable once a diagnosis is established. Difficulties in the clinical conceptualization and management of catatonia have been called "the catatonic dilemma" [1]. Theoretically, how to organize our approach to catatonia remains disputed [2]. Many areas of overlap make the presentation unclear. Even seasoned clinicians do not recognize the often complex presentations of catatonia. The addition or withdrawal of treatments, such as antipsychotic medications, often complicate the clinical picture. As such, many questions in the day to day management of patients persist. Practical social and ethical concerns also interfere with effective treatment at times. The more we learn about catatonia, the more the catatonic dilemma seems to expand. We propose some approaches to detection, classification, and treatment of catatonic syndromes.

### Classification

Since its original description by Karl Kahlbaum in 1874, catatonia has been discussed in the literature and is still actively debated [3]. The DSM-IV TR concept of catatonia, as well as other descriptions, can be confusing as they include some seemingly contradictory clinical signs [2,4]. Motor immobility is described along with excessive motor activity, negativism along with automatic obedience and echopraxia, and mutism along with verbigeration and echolalia. Also, at least forty separate signs of catatonia have been described [2,5]. The reason for this appears to be due to catatonia's truly variable presentation. Peralta et al. found a significant correlation of the excited and retarded motor symptoms, but neither correlated well with psychosis [6]. Some argue that based on the rule of parsimony (Occam's Razor), all of these variable presentations fit into the same category [5]. However, it remains possible that what we now discuss as catatonia may be a nonspecific collection of various illnesses [7,8].

The concept of catatonia has been categorized in various manners related to clinical presentation [2,5]. Catatonia has been conceptualized as chronic or acute, or as isolated acute episodes versus chronic recurrent or periodic catatonia [5,7,9-13]. Catatonia has been divided into retarded and excited forms, the latter often associated with significant progression and morbidity [5,14]. Catatonia has been described in terms of prognosis [2,15]. Catatonia has also been discussed as an independent syndrome versus associated with other psychiatric, neurological, and medical illnesses [2,4,6,8,9,16-18]. Some view catatonia as a "common functional final pathway" in expression of severe neuropsychiatric illness [8]. Taylor and Fink have more recently proposed a classification scheme dividing catatonia into nonmalignant, delirious, and malignant forms, with qualifiers for related illnesses [2]. DSM-IV TR criteria currently requires only two signs, and catatonia is not classified as a separate disorder [4]. A more unified and separate DSM diagnostic criteria set for catatonia will certainly aid in our understanding of its phenomenology, subtypes, comorbidities, natural history, neurophysiology, treatment, and prognosis. A DSM-V classification will also increase psychiatrists' recognition of this common condition, and stimulate further research.

### Epidemiology

One problem in the epidemiologic study of catatonia is that catatonic signs can not be elicited simply by a structured clinical interview, thus it may be under-represented in population samples. Historically, prevalence rates for catatonia have been recorded between 6% and 38% for acute psychotic episodes, and only about 7% to 17% of those patients meet criteria for catatonic schizophrenia [2,5,13]. Catatonic schizophrenia has been reported at a rate of 1 in 1000 in the general population, and up to 5% of all new diagnoses of schizophrenia [11]. Heterogeneous study samples have led to catatonia being described as rare in schizophrenia, and alternately in another study 32% of 225 chronic inpatient schizophrenics met a narrow definition for catatonia [7,18]. Guggenheim and Babigian found that 13% of schizophrenic admissions at a state hospital were of the catatonic subtype, while only 3% of those at a university hospital were catatonic, with a community hospital at an intermediate value [11]. However, multiple other studies of rates of chronic catatonic schizophrenia at single sites, with unchanged definitions over time, show the incidence has decreased significantly (decreases range from 34.2% to 91.7%) over time intervals that correlate with the introduction of neuroleptics [19]. More recently catatonia is described as having a lower incidence of 2% to 8% of acute admissions [8]. Interestingly, the rates for periodic catatonia have remained relatively stable [19]. Thus, decreased incidence of catatonia is not fully explained by changing definitions. Other proposed explanations include improved nursing and social regimes, decreased incidence of viral epidemics coupled with the dying out of a cohort of patients with encephalitis lethargica, a decreased interest by psychiatrists in motor symptoms, and van der Heijden and colleagues have advocated that we are simply under-diagnosing catatonia [18,20-22]. Catatonic symptoms have also been found often in groups of affective disordered patients at rates ranging from 13% to 31%, especially in manic-depressives [2,19,23]. In fact, catatonic signs are not specific to any disorder, and are seen in psychotic disorders, bipolar disorders, depressive disorders, reactive disorders, conversion disorders, dementias, other organic disorders, and without identifiable underlying pathology [8,19]. An accepted and consistent diagnostic scheme for catatonic syndromes will certainly aid in the delineation of their epidemiology.

### Differential diagnosis

The differential diagnosis in catatonia in psychiatric and medical patients can be challenging, and remains a problem for clinicians [9,21]. It is a common occurrence for cases reported in the literature to have the specifics of their diagnosis contested. The differential diagnosis of catatonia involves three parts: (a) recognizing the distinct cluster of signs of a catatonic syndrome; (b) distinguishing catatonia from other movement disorders, including a range of other specific physiological and psychomotor syndromes that may share common features; and (c) identifying sequelae and co-morbidity with other neurologic, medical, and psychiatric pathology. The following discussion focuses mainly on identifying catatonic syndromes presenting in a co-morbid manner with other known neurologic, medical, and psychiatric illnesses.

#### Clinical examination

Given the variability of presentation, and multiple ways of conceptualizing catatonia, a high index of suspicion and broader pattern recognition is needed for the better diagnosis and delineation of catatonic signs and syndromes in medical and psychiatric patients. If a clinician only has in mind the classic description of the mute, cataleptic, rigid, and negativistic schizophrenic they will miss many cases of catatonia. Indeed, there is emerging evidence that catatonic signs are missed in modern clinical practice [21]. Rather than a limited, and often cursory, neurological exam, an active and thorough clinical examination should be part of every acute psychiatric evaluation, especially since patients may exhibit anosognosia [24,25]. Specific catatonic signs to elicit include: complete to semi-elective mutism, stupor, sudden intermittent excitement, echophenomena, stimulus-bound and utilization behavior, stereotypic movements, grimacing and other facial movements, ambitendency, perseveration, mannerisms and other speech disorders, abnormal posture, catalepsy, waxy flexibility, automatic obedience, and negativism [24,26].

#### Neurologic and movement disorders

The ongoing debate over whether the etiology of catatonic syndromes are best understood as psychological or as neurological (e.g., movement disorders) in origin, and complicated by neuroleptic treatment and hospitalization, has been termed a "conflict of paradigms" [8,27,28]. Some advocate viewing catatonia as a form of "psychomotor" disturbance or movement disorder, and since various movement disorders can present similarly, they place emphasis on precise description of motor abnormalities [7,8,28]. Catatonia's immobility, negativism, and waxy flexibility may share features of parkinsonism's bradykinesia and stiffness. Catatonia has many symptoms that overlap with parkinsonism, and cases have been reported of both together [8,9,27,29-31]. Clouding the picture is the use of antipsychotics in the treatment of psychotic disorders. The extrapyramidal side effects of antipsychotics have the same potential for confusion with catatonia as parkinsonism [8,18]. Elderly patients and those with degenerative neurological diseases may present with spontaneous movement disorders, which may be mistaken for catatonia [32,33]. Catatonia shares enough features of neuroleptic malignant syndrome (NMS) that many have examined their relationship [5,34-37]. Distinguishing catatonia from other similar syndromes – malignant hyperthermia, NMS, toxic serotonin syndrome, stiff-person syndrome, locked-in syndrome, hypocalcemia, tetanus, strychnine toxicity, rabies, and elective mutism – has been well-described in the literature [2,5,26,38].

#### Delirium

Catatonia presents commonly in medical settings. Similar to catatonia, delirium has also been described as hypoactive and hyperactive [39,40]. Catatonia can have a waxing and waning presentation, alternating between retardation and excitement, and involve stupor or a change in the level of consciousness [41]. Diagnosis can become difficult, especially in cases with both stupor and mutism, and we need better distinctions [16]. This distinction is particularly important in terms of treatment. Electroconvulsive therapy (ECT) and benzodiazepines can be useful in the treatment of both catatonia and certain forms of delirium, but both may also produce or exacerbate delirium [42-44]. Conversely, antipsychotics used to treat symptoms of delirium have the potential to produce or exacerbate motor signs and catatonia [44-48]. Regardless, a comprehensive evaluation for potential medical causes should be performed in suspected cases of both delirium and catatonia. Underlying medical issues should be addressed for both. With the exception of epileptiform catatonia, relatively normal electroencephalogram and reflex functions may help distinguish catatonic stupor from delirium [49]. In most cases, the diagnosis of delirium will take precedence, and symptoms will gradually clear with treatment of underlying medical illness, and environmental and pharmacologic interventions. In medical catatonia, treatment of the underlying condition commonly does not result in resolution of catatonic symptoms. Therefore, in cases with continued stupor or other catatonic signs, with no clear metabolic, pharmacologic, neurologic or other medical explanation, a diagnosis of catatonia should be considered heavily. At this point, the risk-benefit ratio often favors treatment with a brief trial of benzodiazepines or ECT. We discuss medical co-morbidity and sequelae in more detail later.

#### Psychiatric illness

Similarly, catatonic syndromes often present comorbidly with psychiatric disorders. The periodicity of recurrent catatonic episodes can be reminiscent of bipolar cycling, and others have described a separate entity of confusional cycloid psychosis [6,50-52]. Delirious mania may appear similar to catatonia [37,53]. Also, patients with catatonic excitement may appear hypomanic or manic, though typically with fewer mood symptoms, and more idiosyncratic motor symptoms [54,55]. Some suggest that the presence of catatonic excitement indicates an underlying bipolar disorder [2]. Repetitive mannerisms of catatonia may phenomenologically overlap with obsessive-compulsive symptoms, but the relationship is unclear [18,56,57]. The seemingly volitional nature of negativism can make one question the possibility of malingering, or possibly conversion disorder, if not for the remainder of the clinical presentation.

#### Schizophrenia

A common occurrence in clinical practice is the diffuse and often cavalier use of the diagnosis "chronic paranoid schizophrenia," even amongst trained psychiatrists. This may lead to under-recognition of clinically important catatonic signs in subsequent episodes. It is not uncommon for catatonic schizophrenics to be given other subtype diagnoses during their clinical course, especially of the paranoid subtype [11]. Though this suggests catatonia occurs in relation to schizophrenic episodes and illness, it also allows for the possibility it may be an independent process. Diagnostic subcategories of schizophrenia may be disputed, but were developed with great thoughtfulness, and for a purpose. They are useful not only in communication between clinicians, but have proven helpful in determining potential treatment choices, and prognosis. If a patient clearly has persistent or recurring episodes of catatonia in addition to other psychotic symptoms, the diagnosis of catatonic schizophrenia should be made. The catatonic subtype of schizophrenia in particular has specific treatments that have proven effective, which also suggests it may be a separate entity requiring a unique treatment approach. The catatonic subtype of schizophrenia remains a recognized form of schizophrenia [18,58]. However, the validity of continuing to distinguish a catatonic subtype of schizophrenia requires further study. A separate diagnostic category for catatonia would aid in the recognition and treatment of catatonic syndromes in schizophrenia, and would not necessarily detract from the concepts of schizophrenia.

### Medical comorbidity

Medical conditions, including metabolic and nutritional imbalances have long been associated with catatonia, and it is generally accepted that certain medical conditions can be causative [2,5,9,22,26,59-61]. Studies identify over 35 medical and neurological illnesses associated with catatonia, with those most likely to be causative including CNS structural damage, encephalitis and other CNS infections, seizures, metabolic disturbances, phencyclidine exposure, neuroleptic exposure, lupus cerebritis, corticosteroids, disulfuram, porphyria, and other conditions [61]. Medical catatonia may account for as many as 20% to 30% of cases of catatonia [61]. Distinguishing whether an associated physical finding is a cause or result of catatonia is a difficult process. Many of the findings associated with catatonia may also be a marker for another process, such as poor nutrition or other complications.

#### Laboratory and imaging

Multiple laboratory findings are of interest in catatonic patients, but caution must be exercised in interpreting most of these study results as they are necessarily methodologically limited. Intermittent shifts in nitrogen balance, measured via blood urea nitrogen and urine ammonia or urea, speculatively related to retention of a noxious metabolic substance, have been found to rhythmically correspond to periodic catatonia [62]. Low serum iron and calcium metabolism have been associated with acute or malignant catatonia, especially neuroleptic malignant syndrome (NMS) [63-67]. Elevated white blood cell counts and creatine phosphokinase levels are both nonspecific, though common in acute or malignant catatonia and NMS, appear less common and intense in chronic or simple catatonia [12,62]. Severe hyponatremia and correction has been associated with catatonia [30,31]. Vitamin B_12 _deficiency has been associated with catatonia, and supplementation has resulted in improvement of catatonia [68,69]. Laboratory findings suggesting CNS dysregulation of dopaminergic, noradrenergic, and possibly cholinergic and serotoninergic systems have each been implicated, but require further study along with other neurotransmitters [62]. More recent functional neuroimaging studies have supported the role of the gamma-amino butyric acid (GABA) and glutamatergic (via NMDA-receptors) systems in catatonia [8]. Computed tomography studies in catatonic patients have reported cortical enlargement and cerebellar atrophy, but no consistently specific findings [8].

#### Medical sequelae

Medical complications are common in catatonia, even "benign" catatonia [22,60,70]. Psychotic and other mentally ill patients often have unhealthy lifestyles, may be unable to care properly for their medical conditions, and are at an increased risk for developing various complications [71,72]. Another common clinical concern is that catatonic behavior can mask signs and symptoms of serious underlying medical conditions, necessitating vigilance in ongoing physical evaluation of catatonic patients [11]. Patients with catatonic disorders who are unable to care for their own medical problems are often neglected in chronic institutionalized settings. Chronic catatonic patients are at increased risk for developing aspiration, pulmonary embolus, drug-resistant infections, dehydration, malnutrition necessitating enteral feeding, constipation, contractures, skin breakdown, poor dentition, poor menstrual hygiene, urinary retention and bladder infections [73]. Prophylactic measures should be carefully considered as they often put the patient at risk for other complications. There is a continued need to develop treatment methods, beyond partial response in catatonia and schizophrenia, to improve the quality of life for our patients.

### Cognition, emotion and behavior

#### Phenomenology

The complex interactions between cognition, emotion and behavior in catatonia will eventually lead to better understanding of its etiology. Kahlbaum's original conception described catatonia as part of generally deteriorating mental process [22,74]. Defective memory was common (56 of 214) in another large sample of catatonic schizophrenics [15]. Catatonia has been reported to result in lasting cognitive impairment [75]. Neuropsychological testing on recently recovered catatonic patients has preliminarily revealed intact general intelligence, attention and executive functions, deficits in right parietal visuo-spatial function and emotionally guided intuitive decisions on gambling tasks, and disturbed constructs of "self" [8]. Phenomenologically, recovered catatonic patients usually describe having experienced intense emotional states, often uncontrollable anxiety and overwhelming fear, but also ambivalence, depression, euphoria, lability, aggression and psychosis [8,76]. Motor signs are common in patients with psychotic, mood and anxiety disorders, but behavior and motor disturbances in catatonic patients may be our only evidence to guide diagnostic and treatment decisions [26,77]. Frequently catatonics remain unaware of their behavioral and motor signs, reporting that they were functioning normally. Catatonic patients may exhibit behavior that appears consciously produced, though many of the cognitive processes reflected in catatonic behavior likely occur unconsciously [78].

#### Recent theories

As a complex psychomotor syndrome, catatonia has been conceptualized by Northoff as a predominantly a cortical process dynamically interacting with subcortical regions, involving cognitive, emotional, and behavioral circuits [8,79,80]. Northoff distinguishes catatonia from parkinsonism and NMS, which he describes as mainly subcortical syndromes [80]. Northoff hypothesizes that, in catatonic patients, alterations primarily in orbitofrontal and right posterior parietal functioning may modulate other cortical structures responsible for cognition, affect, and behavior, as well as subcortical structures responsible for movement [79,80]. A hyperdopaminergic mesolimbic system may attempt a restitutive downregulation of dopamine to ward off psychosis, thus affecting other systems, and producing motor symptoms via the nigrostriatal pathway [62,81]. Involvement of the anterior cingulate, amygdala, hippocampi, thalamic nuclei and basal ganglia are possible. Fricchione also conceptualizes catatonia in a complex network model of neurocircuits and loops at various levels, hypothesizing a disorder of basal ganglia thalamo(limbic)-cortical circuits [81]. However, Fricchione focuses on explaining catatonia in terms the evolution of structures and circuits responsible for mammalian social behavior, specifically attachment-separation adaption strategies [81]. Catatonic syndromes may represent a regression to or neurological vestige of more primitive mechanisms, modulating disinhibited confrontation versus freezing strategies for dealing with threat, which would necessarily be linked to intense emotions [76,78,81,82]. These models allow for production of catatonic signs and syndromes from dysfunction of various neurotransmitter systems or neurological lesions. To further explore these issues, a consensus is needed on how to best measure and approach both cognition and affect in catatonic patients, who are by the nature of their illness limited in their ability to cooperate and report symptoms.

### Treatment

#### Benzodiazepines

An initial trial of pharmacotherapy is often possible and preferable in catatonic patients. Historically, a trial of sodium amytal was recommended [83]. More recently, benzodiazepines, especially lorazepam from modest to high doses, have been widely studied and used in the treatment of the various forms of catatonia, but most studies are small and have methodological shortcomings [2,84-87]. Though this has been proven to be a good treatment on average, the degree of symptom response for individual patients may vary tremendously. At least one clinical trial has suggested that lorazepam may not be efficacious in the treatment of chronic catatonia [88]. Another concern with use of benzodiazepines in the case of poor or non-response is that catatonia has also been reported with benzodiazepine withdrawal [89,90]. Thus, a trial of a benzodiazepine such as lorazepam to test for response should be attempted, especially in the retarded form of catatonia, but if there is no initial response at an adequate dose the trial should be kept brief to avoid interference with ECT and the possibility of exacerbating the syndrome via withdrawal. Earlier consideration of ECT could avert such a dilemma.

#### Antipsychotics and other medications

The question remains whether or not to use antipsychotics in catatonia, especially newer agents in nonmalignant catatonic patients with a history of psychosis or diagnosis of a psychotic disorder [45]. A concern is causing worsening in symptomatology with neuroleptic treatment, or clouding the clinical picture with parkinsonism [34,46-48,91,92]. Antipsychotics have been reported to cause neuroleptic-induced catatonia [46,48]. Antipsychotics, even the atypicals, are known to induce neuroleptic malignant syndrome in catatonic patients [9,46,93-95]. One study found no correlation between antipsychotic dose and severity of catatonia [18]. However, rates of catatonic schizophrenia have decreased since the introduction of neuroleptics [19]. Psychotropics that have been suggested and studied in the treatment of catatonia – including clozapine, other atypical antipsychotics, anticonvulsants, lithium, amantadine and others – have shown variable degrees of success [8,96-101]. Variable response may reflect responses to the wide array of comorbid conditions seen with catatonic syndromes more than a specific effect on catatonia itself. If catatonia is to be viewed as a distinct syndrome that may be comorbid and interact with other disorders, concomitant treatment of syndromes should be attempted. It is reasonable to attempt a concomitant trial of atypical antipsychotics if the patient has a history of chronic psychotic illness or exhibits psychotic signs or symptoms in addition to catatonic signs. Avoid higher potency antipsychotics, and monitor closely for adverse effects, especially NMS. Similarly, a trial of an antidepressant or mood stabilizer treatment would be indicated for catatonia in the setting of a mood episode or disorder.

#### Electroconvulsive therapy

Clinicians are often left with the question of what to do in the case of poor to partial response to initial pharmacological treatment. Though some report the high response rates of catatonia, partial responses to benzodiazepine therapy is common, especially in catatonic schizophrenia [5,9]. Always reconsider the diagnosis. ECT is considered the ultimate treatment for catatonia, especially resistant forms [8,42,43]. ECT has been shown effective in the treatment in cases of prolonged catatonia, even those partially responsive to pharmacologic treatments [102,103]. Poor nutrition and other complications are often the deciding factors in whether to pursue ECT or not. However, practical, cultural, ethical and legal considerations determine how far to push for such treatment, and patients or guardians are often reluctant to consent to ECT, despite education [104-106]. Because of the stigma and past abuses, and remaining controversy about potential adverse effects, there are multiple legal and ethical barriers to the use of ECT, which can be frustrating to providers who wish to provide a treatment known to be safe and effective for patients with grave disability due to catatonia [107]. Each decision must be tailored to the individual patient's situation. Regardless, given its effectiveness and tolerability, clinicians should advocate early for acceptance of ECT treatment in cases of catatonia.

#### Psychosocial treatments

Despite a history of being viewed by Kraepelin and Bleuler as originating from psychic factors, or by others as a social construct, there is currently a dearth of information on practical psychosocial and environmental treatments in catatonic syndromes [8,22,28]. In fact, some have argued that interventions such as hospitalization may worsen catatonia [28]. Catatonia is now viewed by most clinicians in a predominantly biologic framework. We do not know the possible role of intense emotional states in perpetuating or predisposing to catatonia. However, a more complete biopsychosocial model has been helpful in understanding and treating catatonia [76,108]. Though purely psychoanalytic treatments may be considered failed attempts by many, their contribution to the field is undeniable [22]. More recent neuroanatomic theories in many ways parallel earlier psychoanalytic theories [78,82]. Psychological and social interventions should complement biological treatments, and may have particular utility as an adjunct in cases of chronic catatonia, or in those with poor treatment response. Certainly, we need more research in this area.

#### Treatment response

Catatonia remains a clinical diagnosis. Thus, treatment response may be difficult to objectively measure. Though typically less well-validated or specific to a co-morbid disorder, after diagnosis is established, briefer versions of rating scales can be useful clinically in monitoring treatment response in patients with catatonia [109-111]. Of these, the Bush Francis Catatonia Rating Scale is currently the most sensitive and best validated rating instrument for broad clinical use with acute catatonic patients [109]. One should also consider the Rogers catatonia scale for use in patients with depressive and other mood disorders [110]. Also, consider monitoring creatine phosphokinase levels in cases of acute catatonia.

### Prognosis

From the earliest descriptions of catatonia it was noted to have an extremely variable prognosis, ranging from good recovery to acute onset with rapid progression to death (often complicated by renal failure) [73]. Most likely, the greatest factor in determining prognosis in a catatonic syndrome is the nature and severity of comorbid or underlying conditions [73]. Studies thus reflect that prognosis in catatonic syndromes may in part depend on the definition used, and opinions differ widely [112]. In Kahlbaum's syndrome, which also included mood episodes, prognosis was better than for schizophrenia [6]. Recurrent or periodic catatonia has been associated with a very good prognosis [73]. Affective and alcohol use disorders, as well as diagnosis with a reactive psychosis or acute illness, have been shown predictive of better response [15,112]. Relationship of specific motor symptoms to response rates has been examined in catatonic schizophrenics, but consistent results have not been reported in the literature [15,23]. The presence of catatonia in schizophrenics has been associated with earlier and greater mortality [11,113]. Ungvari et al found a high proportion of catatonia among institutionalized schizophrenics [18]. Severity of catatonia has been correlated with poorer functional outcome [18]. Levenson and Pandurangi nicely reviewed relative prognosis by associated condition from best to worst as: mood disorder without catatonia, depression with catatonia, periodic catatonia, cycloid psychoses with catatonia, bipolar disorder with catatonia, catatonic schizophrenia, then non-catatonic schizophrenia [73]. Another factor that affects prognosis is which treatments are used, which vary across treatment settings, and have varied throughout the years. Younger age at onset, confusion and diagnosis of schizophrenia have been shown predictive of poorer treatment response [15,112]. Some studies suggest catatonic schizophrenia is only 40% to 50% likely to respond to benzodiazepines [9,114]. Biological markers have not yet been consistently associated with prognosis and require further study [73].

### Further study

Subsets of patients and families with specific forms of catatonia have been studied since the 1950s [15]. Stöber and colleagues discovered the first specific gene to be associated with a primary psychiatric disorder while studying periodic catatonia [115-119]. There is some evidence that certain patients may have a genetic predisposition to development of neuroleptic malignant syndrome with antipsychotic treatment during catatonia [120]. Further genetic discoveries are inevitable. Whether catatonic signs and behavior constitute a specific subtype of schizophrenia, versus separate but often comorbid conditions, needs further study if catatonic signs are continued to be included in the diagnostic criteria for schizophrenia. Catatonic syndromes should also be included in studies of delirium. Functional neuroimaging offers promise in the study of catatonia [79,80,121]. More neuropathologic studies are also needed. With time, translational research may bring more strategies for diagnosis, to help determine response to various treatment options, and elucidate other prognostic factors that may assist in management. Genetic and imaging research may also lend insight into the pathophysiology of the various clinical presentations of catatonia, though this could take decades. Now, with the advent of well-validated and comprehensive catatonia rating scales, research in catatonia will have added objectivity [122,123]. We especially need further developments and controlled pharmacologic and psychosocial treatment trials in all forms of catatonia. Transcranial magnetic stimulation (TMS) is not currently a valid treatment, but if catatonia turns out to be a predominantly cortical process then there may be a role for it [124,125]. Perhaps TMS and other somatic treatments may eventually prove beneficial in catatonia.

## Conclusion

It is clear from the review of literature that we need a better consensus and greater clarity in the diagnosis and description of catatonia. A high index of suspicion and broader syndrome pattern recognition for catatonia should be exercised by clinicians, and should include precise description of signs. Better recognition will help us better delineate catatonic syndromes. By expanding our nosology with genetic and other translational basic science research we can better understand the pathophysiology of catatonia. A separate and more descriptive DSM-V diagnostic criteria set for catatonia will aid in our understanding of catatonia's phenomenology, subtypes, comorbidities, natural history, neurophysiology, treatment and prognosis. An improved DSM-V classification will also increase psychiatrists' recognition of this common syndrome, and stimulate further research. We will likely gain better understanding of the intricacies of psychotic, mood, movement, anxiety, and somatoform disorders, as well as such issues as volition and will. This will lead to delineation of prognosis, better management choices, and ultimately to improvement in the general medical health and quality of life of our patients. Practical roadblocks and ethical concerns often get in the way of treatment. We must continue to advocate for better social acceptability of electroconvulsive therapy as a legitimate treatment option in psychiatric and medical patients. Meanwhile, we must continue to attend to the psychological and social needs of catatonic patients.

## Declaration of competing interests

"This project is supported by funds from the Division of State, Community, and Public Health, Bureau of Health Professions (BHPr), Health Resources and Services Administration (HRSA), Department of Health and Human Services (DHHS) under grant number 1 K01 HP 00071-03 and Geriatric Academic Career Award. The information or content and conclusion are those of the author – Rajesh R. Tampi M. D., M. S., and should not be construed as the official position or policy of, nor should be any endorsements be inferred by the Bureau of Health Professions, HRSA, DHHS or the U.S. Government."
